# Supplementary material for: Structural basis of catalytic activation in human splicing
Source: Nature. 2023 May 10;617(7962):842–50. doi: 10.1038/s41586-023-06049-w (PMC10208982; doi:10.1038/s41586-023-06049-w)
Supplement: Supplementary file 1 — Supplementary figures and legends for the supplementary figures, the supplementary data files and the supplementary video files. [file 41586_2023_6049_MOESM1_ESM.pdf]

nature portfolio

https://doi.org/10.1038/s41586-023-06049-w

Supplementary information

Structural basis of catalytic activation in human splicing

In the format provided by the authors and unedited

SI Guide

Structural basis of catalytic activation in human splicing

Jana Schmitzová[1†‡], Constantin Cretu[1,2§‡], Christian Dienemann[3], Henning Urlaub[4], Vladimir Pena[1,2*]

‡ These authors contributed equally to this work

* Correspondence

Authors Affiliations

[1]Macromolecular Crystallography, Max Planck Institute for Multidisciplinary Sciences, Göttingen, Germany

[2]Research Group Mechanisms and Regulation of Splicing, The Institute of Cancer Research, London, United Kingdom

[3]Molecular Biology, Max Planck Institute for Multidisciplinary Sciences, Göttingen, Germany

[4]Bioanalytical Mass Spectrometry, Max Planck Institute for Multidisciplinary Sciences, Göttingen, Germany; Institute of Clinical Chemistry, Bioanalytics, Institute of Clinical Chemistry, University Medical Center Göttingen, Germany

†Present address: Molecular Biology, Max Planck Institute for Multidisciplinary Sciences, Göttingen, Germany

§Present address: Cluster of Excellence Multiscale Bioimaging (*MBExC*), Universitätsmedizin Göttingen, Göttingen, Germany

Correspondence to be addressed to V.P. at: vlad.pena@icr.ac.uk

Table of Content:

SI Figure 1 | Uncropped gels from Extended Data Figure 1. **a**, Pre-mRNA splicing is arrested by incorporating the recombinant dominant-negative IBC/Aquarius^{K829A} in the human spliceosomes (left), while the wild-type IBC does not influence splicing (right). The Cy5-labeled RNA substrate was analyzed on a denaturing polyacrylamide gel and visualized by in-gel fluorescence. The cropped area shown in the Extended Data Figure 1b is outlined. **b-c,** The protein composition of the B^{AQR} complex purified via MBP-MS2 affinity (b) followed by gradient ultracentrifugation (c). The SDS-PAGE gels were stained with Coomassie. **d,** RNA composition of the purified B^{AQR} complexes. The SDS-PAGE gel was stained with SYBR Gold. The cropped areas shown in Extended Data Figure 1c are outlined.

SI Figure 2 | Uncropped SDS-PAGE protein gels from Extended Data Figure 7. a,b, PRP2 forms stable complexes with PPIL4 and GPKOW. The reconstitution of the PRP2 (137-1022)-GPKOW and PRP2 (137-1022)-PPIL4 complexes was assessed by size-exclusion chromatography (SEC) on the Superdex 200 column and the peak fractions analyzed by SDS-PAGE. The SEC fraction numbers of the analyzed samples are indicated above the SDS-PAGE gels. The shift in PRP2's peak fraction (fraction 11) in the presence of both PPIL4 and GPKOW is consistent with stable complex formation, independent of RNA. The cropped areas shown in Extended Data Figures 7b and 7f are outlined. **c,** Reconstitution of a stable PRP2-PPIL4 complex by *in vivo* co-expression in insect cells. PRP2 (137-1022) and PPIL4 were co-expressed in Sf9 insect cells from individual baculoviruses and their complex was captured on Strep-Tactin affinity beads followed by SEC on a Superdex 200 column. The gel corresponding to the SEC fractions is shown (left) and the comparison of the SEC chromatograms corresponding to the complex obtained in vivo and in vitro is depicted (right). The cropped area and shown in Extended Data Figure 7c is outlined.

SI Figure 3 | Uncropped RNA gels from Extended Data Figure 7. The legend of the supplementary figures are the same as in the indicated Extended Data figures.

SI Figure 4 | Sequence alignment of PRP2, SKIP, and PPIL4 orthologs. The sequences of $PRP2^{NTD}$, the molecular brake's SKIP region, and the full-length PPIL4 are shown. *Hs – Homo sapiens, Ce – Caenorhabditis elegans, At – Arabidopsis thaliana, Sp – Schizosaccharomyces pombe, Sc – Saccharomyces cerevisiae.*

SI Video 1. Overview of the cryo-EM density of the B^{AQR} spliceosome. The overall density is shown in grey and the densities of the core and peripheric modules are colored.

SI Video 2. Translocation of PRP2 along the intron and SF3B1's opening on the branch duplex, at the transition from B^{act} to B^{AQR} complexes.

SI Video 3. Remodeling of the spliceosome by PRP2's translocation, at the B^{act} to B^{AQR} transition. The movie depicts the stepwise destabilization of subunits followed by the assembly of the molecular brake that terminates PRP2's translocation.

SI Video 4. Remodeling of B^{AQR} at the transition to B^{*}/C complexes. The movie depicts the destabilization of various subunits present in B^{AQR} and the relocation of the branch duplex to the active center of the spliceosome for the branching reaction.

SI Video 5. Termination of PRP2's translocation by the construction of a molecular brake. PPIL4, SKIP, and $PRP2^{NTD}$ bind the exiting intron-like a shoe brake, while SKIP's wedge element intercalates between the RecA-like and CTD domains of PRP2, immobilizing the helicase in the open conformation. PRP2 in the open conformation (after ATP hydrolysis and translocation) corresponds to the B^{AQR} structure. The closed conformation is modeled based on the crystal structure of Prp2 from *Chaetomium thermophillum*, in complex with ADP-$BeF3^{-}$ and a poly-U_{12} RNA (PDB 6zm2).

Caption for Data S1. Protein composition of purified human B^{AQR} spliceosomes as revealed by mass spectrometry analyses.

Caption for Data S2. Model building details for the B^{AQR} complex. The B^{AQR} subunits/regions modeled only in the overall maps M5 and M4 (corresponding to states A and B) are colored in purple.

Figure 1

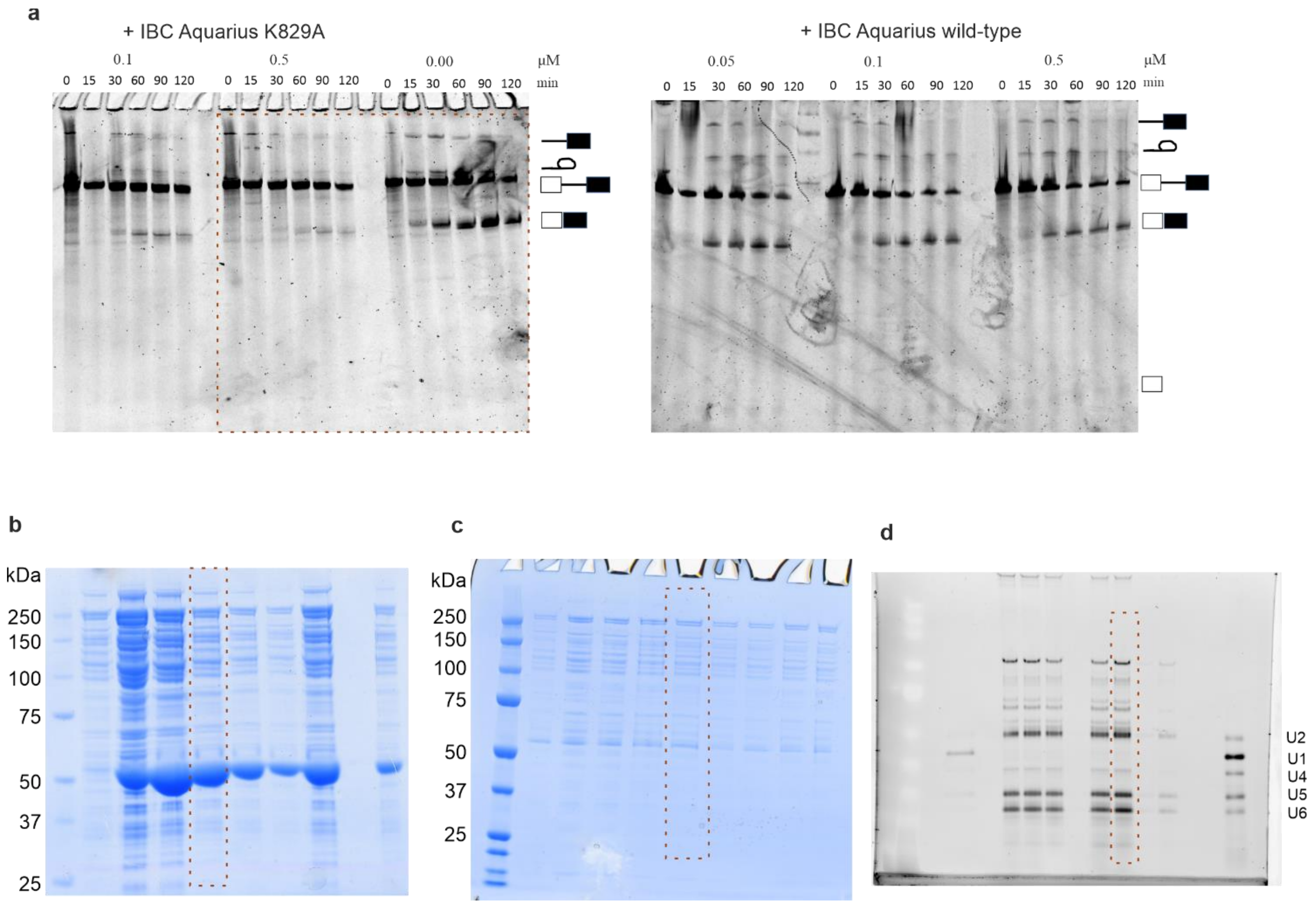

a
+ IBC Aquarius K829A
0.1
0.5
0.00
µM
0 15 30 60 90 120 0 15 30 60 90 120 0 15 30 60 90 120 min
+ IBC Aquarius wild-type
0.05
0.1
0.5
µM
0 15 30 60 90 120 0 15 30 60 90 120 0 15 30 60 90 120 min
b
kDa
250
150
100
75
50
37
25
c
kDa
250
150
100
75
50
37
25
d
U2
U1
U4
U5
U6

Figure 2

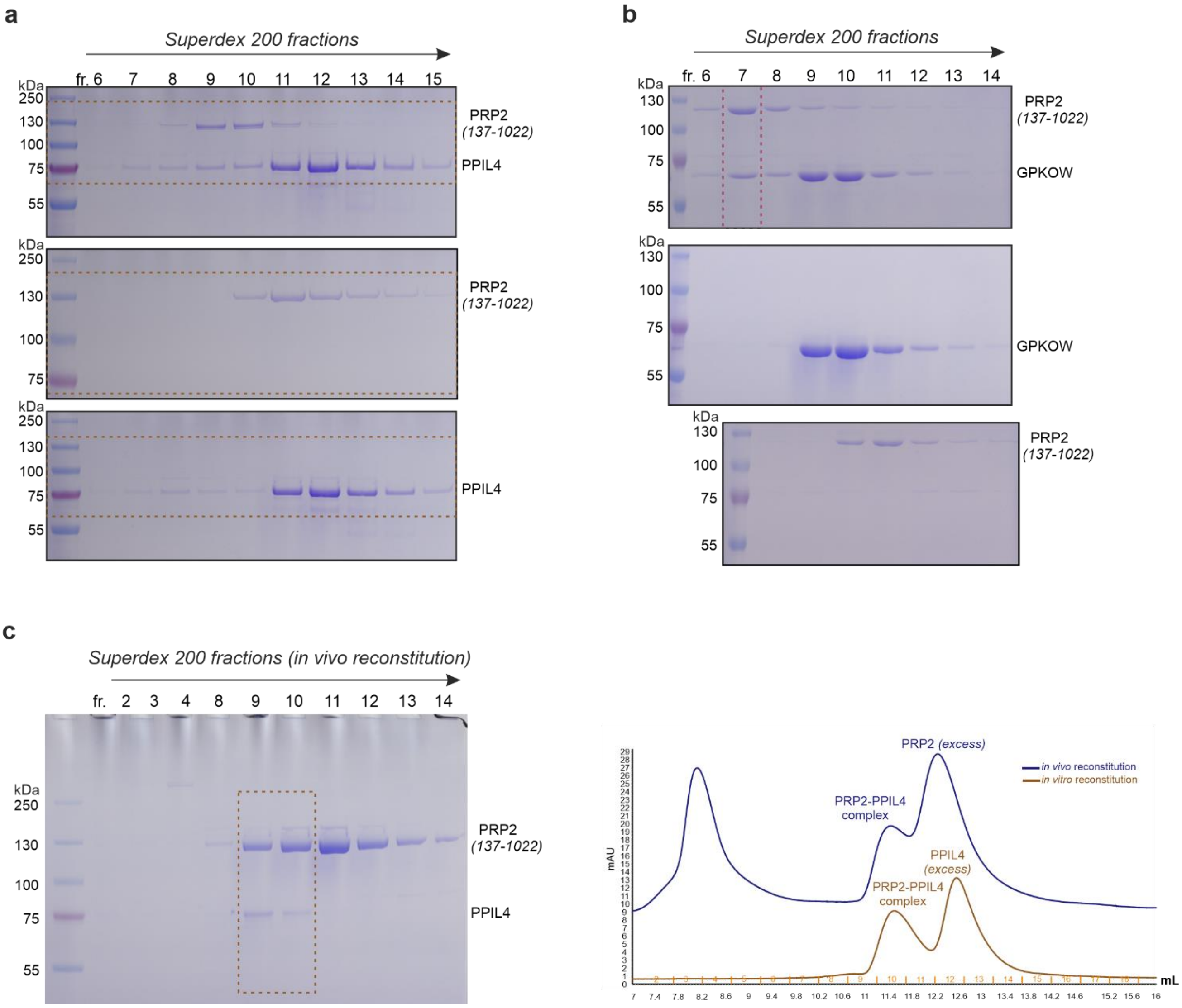

a
Superdex 200 fractions
kDa
250
130
100
75
55
fr. 6
7
8
9
10
11
12
13
14
15
PRP2
(137-1022)
PPIL4
b
Superdex 200 fractions
GPKOW
c
Superdex 200 fractions (in vivo reconstitution)
fr.
2
3
4
PRP2 (excess)
in vivo reconstitution
in vitro reconstitution
PRP2-PPIL4
complex
PPIL4
(excess)
mAU
mL

Figure 3

Extended Data Figure 7d

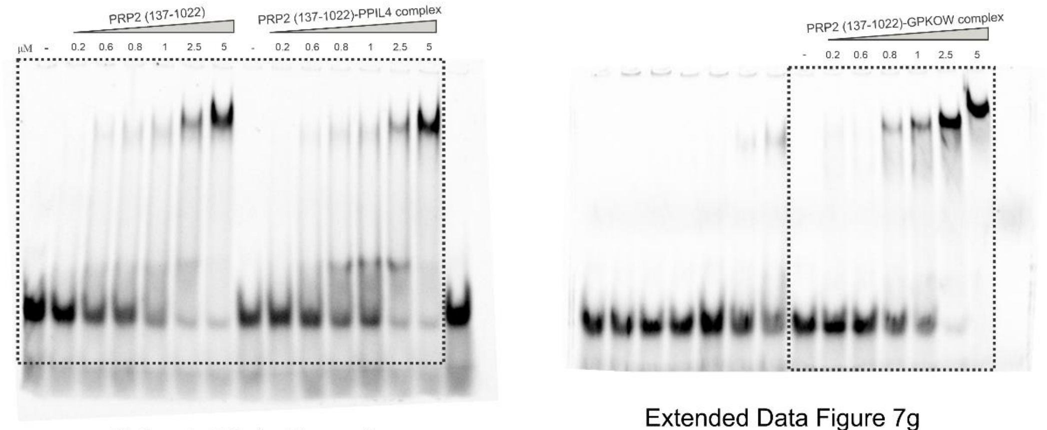

Extended Data Figure 7e

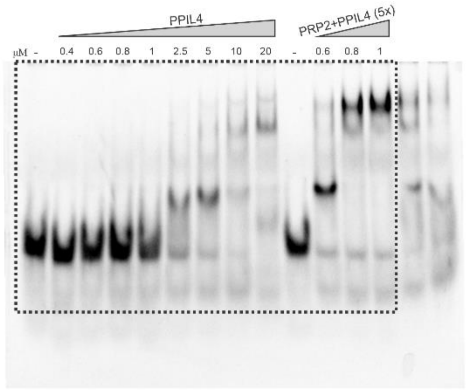

Extended Data Figure 7g

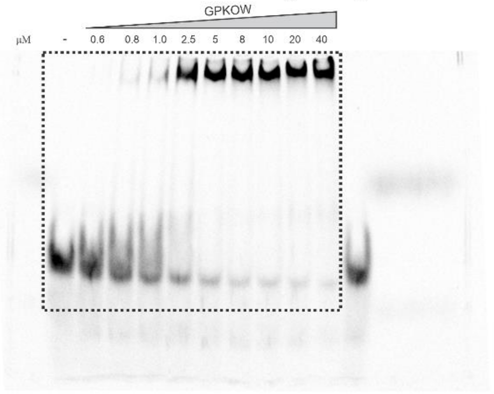

Extended Data Figure 7i

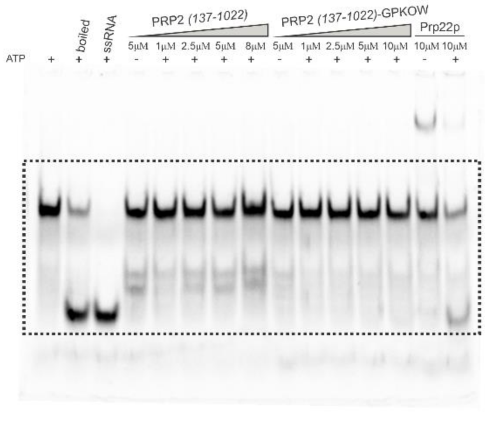

Extended Data Figure 7j

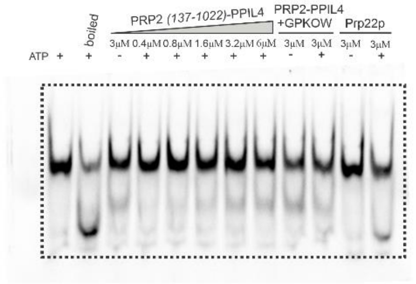

Extended Data Figure 7k

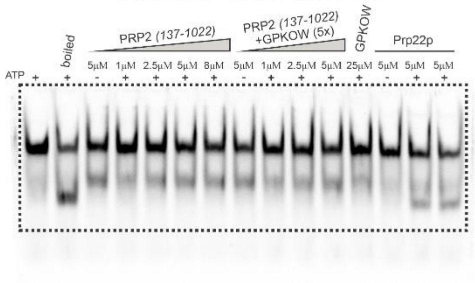

Extended Data Figure 7l

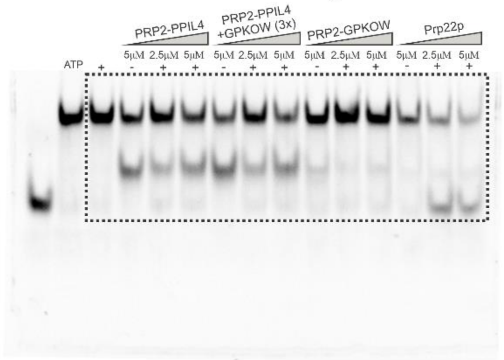

Figure 4

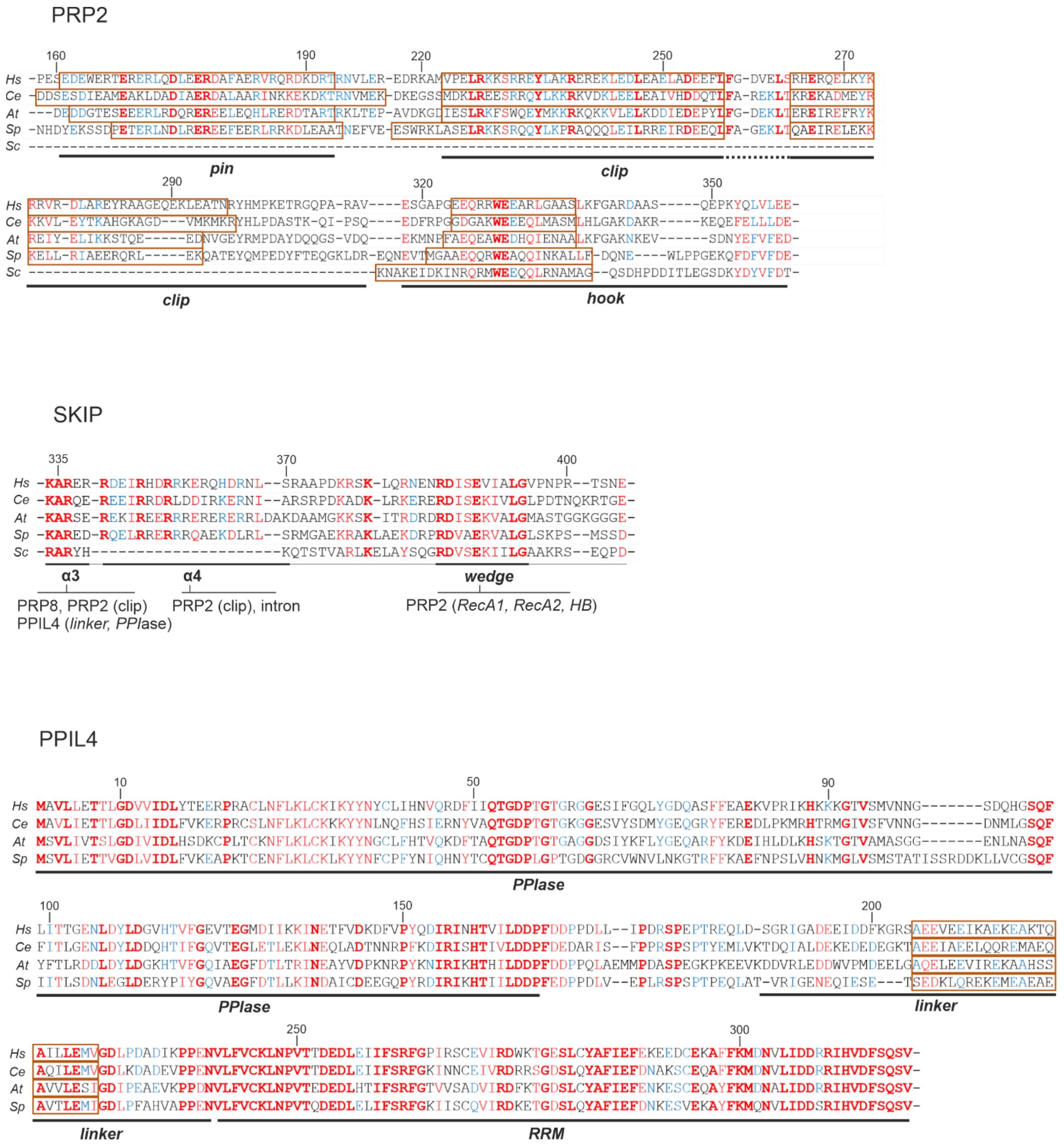
PRP2
pin
clip
hook
SKIP
α3
α4
wedge
PRP8, PRP2 (clip)
PPIL4 (linker, PPIase)
PRP2 (clip), intron
PRP2 (RecA1, RecA2, HB)
PPIL4
PPIase
linker
RRM
