# Supplementary material for: Structural basis of catalytic activation in human splicing
Source: Nature. 2023 May 10;617(7962):842–50. doi: 10.1038/s41586-023-06049-w (PMC10208982; doi:10.1038/s41586-023-06049-w)
Supplement: Supplementary file 2 — Reporting Summary [file 41586_2023_6049_MOESM2_ESM.pdf]

Corresponding author(s): Vladimir Pena
Last updated by author(s): Mar 9, 2023

Reporting Summary

Nature Portfolio wishes to improve the reproducibility of the work that we publish. This form provides structure for consistency and transparency in reporting. For further information on Nature Portfolio policies, see our Editorial Policies and the Editorial Policy Checklist.

Statistics

For all statistical analyses, confirm that the following items are present in the figure legend, table legend, main text, or Methods section.

n/a	Confirmed	
☐	☒	The exact sample size (*n*) for each experimental group/condition, given as a discrete number and unit of measurement
☒	☐	A statement on whether measurements were taken from distinct samples or whether the same sample was measured repeatedly
☒	☐	The statistical test(s) used AND whether they are one- or two-sided<br>*Only common tests should be described solely by name; describe more complex techniques in the Methods section.*
☒	☐	A description of all covariates tested
☒	☐	A description of any assumptions or corrections, such as tests of normality and adjustment for multiple comparisons
☒	☐	A full description of the statistical parameters including central tendency (e.g. means) or other basic estimates (e.g. regression coefficient) AND variation (e.g. standard deviation) or associated estimates of uncertainty (e.g. confidence intervals)
☒	☐	For null hypothesis testing, the test statistic (e.g. *F*, *t*, *r*) with confidence intervals, effect sizes, degrees of freedom and *P* value noted<br>*Give P values as exact values whenever suitable.*
☒	☐	For Bayesian analysis, information on the choice of priors and Markov chain Monte Carlo settings
☒	☐	For hierarchical and complex designs, identification of the appropriate level for tests and full reporting of outcomes
☒	☐	Estimates of effect sizes (e.g. Cohen's *d*, Pearson's *r*), indicating how they were calculated

Our web collection on statistics for biologists contains articles on many of the points above.

Software and code

Policy information about availability of computer code

Data collection	The cryo-EM data were collected using EPU on a Titan Krios G2 electron microscope operated at 300 keV in EFTEM mode and equipped with a Quantum LS 967 energy filter (Gatan) and a K2 Summit direct electron detector (Gatan).
Data analysis	CCP4 (v. 7.0 .045), CCPEM (v. 1.4.1), Phenix (v. 1.19-4092), Coot (v. 0.9.6), UCSF Chimera (v. 1.14, v. 1.16), ChimeraX (v. 1.3, v. 1.5), Warp, cryoSPARC v0.65, RELION 3.0, RELION 3.1-beta, Pymol (v. 2.4.1), GraphPad Prism 9 (v. 9.5.0), OriginPro 2020 (v. 9.7), DeepEMhancer, Alphafold 2, ColabFold (v.1.5)

For manuscripts utilizing custom algorithms or software that are central to the research but not yet described in published literature, software must be made available to editors and reviewers. We strongly encourage code deposition in a community repository (e.g. GitHub). See the Nature Portfolio guidelines for submitting code & software for further information.

Data

Policy information about availability of data

All manuscripts must include a data availability statement. This statement should provide the following information, where applicable:

- Accession codes, unique identifiers, or web links for publicly available datasets
- A description of any restrictions on data availability
- For clinical datasets or third party data, please ensure that the statement adheres to our policy

The structural models and cryo-EM maps of the BAQR spliceosome (the core region and the state B holocomplex) were deposited in the Protein Data Bank (PDB accession codes 7QTT and 8CH6) and Electron Microscopy Data Bank (accession codes EMD-14146 amd EMD-16658), respectively.

Human research participants

Policy information about studies involving human research participants and Sex and Gender in Research.

Reporting on sex and gender	*Use the terms sex (biological attribute) and gender (shaped by social and cultural circumstances) carefully in order to avoid confusing both terms. Indicate if findings apply to only one sex or gender; describe whether sex and gender were considered in study design whether sex and/or gender was determined based on self-reporting or assigned and methods used. Provide in the source data disaggregated sex and gender data where this information has been collected, and consent has been obtained for sharing of individual-level data; provide overall numbers in this Reporting Summary. Please state if this information has not been collected. Report sex- and gender-based analyses where performed, justify reasons for lack of sex- and gender-based analysis.*
Population characteristics	*Describe the covariate-relevant population characteristics of the human research participants (e.g. age, genotypic information, past and current diagnosis and treatment categories). If you filled out the behavioural & social sciences study design questions and have nothing to add here, write "See above."*
Recruitment	*Describe how participants were recruited. Outline any potential self-selection bias or other biases that may be present and how these are likely to impact results.*
Ethics oversight	*Identify the organization(s) that approved the study protocol.*

Note that full information on the approval of the study protocol must also be provided in the manuscript.

Field-specific reporting

Please select the one below that is the best fit for your research. If you are not sure, read the appropriate sections before making your selection.

[X] Life sciences [ ] Behavioural & social sciences [ ] Ecological, evolutionary & environmental sciences

For a reference copy of the document with all sections, see nature.com/documents/nr-reporting-summary-flat.pdf

Life sciences study design

All studies must disclose on these points even when the disclosure is negative.

Sample size	Sample-size calculation was not performed. All biological/biochemical experiments were replicated two or more times. Two cryo-EM datasets of the BAQR spliceosome were collected at 0 and 25 tilt angles.
Data exclusions	Contaminated and low-resolution cryo-EM micrographs were excluded from the analysis with Warp.
Replication	All attempts at replication were successful.
Randomization	No randomization was required.
Blinding	Blinding is not applicable for the methods used in this study.

Reporting for specific materials, systems and methods

We require information from authors about some types of materials, experimental systems and methods used in many studies. Here, indicate whether each material, system or method listed is relevant to your study. If you are not sure if a list item applies to your research, read the appropriate section before selecting a response.

March 2021

Materials & experimental systems

n/a	Involved in the study
☒	☐ Antibodies
☐	☒ Eukaryotic cell lines
☒	☐ Palaeontology and archaeology
☒	☐ Animals and other organisms
☒	☐ Clinical data
☒	☐ Dual use research of concern

Methods

n/a	Involved in the study
☒	☐ ChIP-seq
☒	☐ Flow cytometry
☒	☐ MRI-based neuroimaging

Eukaryotic cell lines

Policy information about cell lines and Sex and Gender in Research

Cell line source(s)	High Five (Trichoplusia Ni, BTI-Tn-5B1-4) insect cells were cultured in ESF921 media and obtained from ThermoFisher Scientific, Cat#B85502 or Expression Systems, Cat#94-002F<br>Sf9 (Spodoptera frugiperda) insect cells were cultured in Sf-900 III SFM and obtained from ThermoFisher Scientific, Cat#11496015 and Cat#12659017.<br>HeLa S3 cells, used for nuclear extract preparation, were obtained from Helmoltz Center for Infection Research, Brunswick.
Authentication	Insect cell lines were not authenticated.
Mycoplasma contamination	Insect cell lines were not tested for Mycoplasma contamination. HeLa S3 cells were tested for Mycoplasma contamination.
Commonly misidentified lines (See ICLAC register)	No commonly misidentified cell lines were used.
